# Targeting β-Catenin Signaling by Natural Products for Cancer Prevention and Therapy

**DOI:** 10.3389/fphar.2020.00984

**Published:** 2020-06-30

**Authors:** Wen-Kai Yu, Zhi-Yuan Xu, Li Yuan, Shaowei Mo, Beihua Xu, Xiang-Dong Cheng, Jiang-Jiang Qin

**Affiliations:** ^1^ College of Pharmaceutical Sciences, Zhejiang Chinese Medical University, Hangzhou, China; ^2^ Institute of Cancer and Basic Medicine, Chinese Academy of Sciences, Cancer Hospital of the University of Chinese Academy of Sciences, Zhejiang Cancer Hospital, Hangzhou, China; ^3^ The First Affiliated Hospital of Zhejiang Chinese Medical University, Hangzhou, China

**Keywords:** β-catenin, cancer prevention and therapy, degradation, inhibitors, natural products, phosphorylation

## Abstract

The mutations and deregulation of Wnt signaling pathway occur commonly in human cancer and cause the aberrant activation of β-catenin and β-catenin-dependent transcription, thus contributing to cancer development and progression. Therefore, β-catenin has been demonstrated as a promising target for cancer prevention and therapy. Many natural products have been characterized as inhibitors of the β-catenin signaling through down-regulating β-catenin expression, modulating its phosphorylation, promoting its ubiquitination and proteasomal degradation, inhibiting its nuclear translocation, or other molecular mechanisms. These natural product inhibitors have shown preventive and therapeutic efficacy in various cancer models *in vitro* and *in vivo*. In the present review, we comprehensively discuss the natural product β-catenin inhibitors, their *in vitro* and *in vivo* anticancer activities, and underlying molecular mechanisms. We also discuss the current β-catenin-targeting strategies and other potential strategies that may be examined for identifying new β-catenin inhibitors as cancer preventive and therapeutic drugs.

## Introduction

β-Catenin was initially discovered as one of the E-cadherin-associated molecules on the cell membrane and the E-cadherin/β-catenin complex contributes to Ca^2+^-dependent cell-cell adhesion (Ozawa et al., 1989; Ozawa et al., 1990). β-Catenin was found to play an essential role in the embryonic development of *Drosophila melanogaster* (Wieschaus et al., 1984). In the saturation mutagenesis screens for identifying genes that regulate the segmentation of *Drosophila* embryo, the *Drosophila* ortholog of the mammalian β-catenin gene (*CTNNB1*), termed *armadillo* has been found to affect the formation of denticles and naked belts (Wieschaus et al., 1984). Further analysis has characterized the Wnt/β-catenin pathway by demonstrating the critical role of Wnt proteins in activating the β-catenin signaling pathway (Riggleman et al., 1990). In the canonical Wnt/β-catenin signaling pathway, β-catenin functions as a coactivator of the transcription factor T cell factor/lymphocyte enhancer factor (TCF/LEF) and promotes the transcription of Wnt target genes, which are responsible for controlling cell fate in various diseases, including cancer (Cui et al., 2018).

β-Catenin is overexpressed and constitutively activated in human cancer and contributes to cancer initiation, progression, metastasis, drug resistance, and immune evasion (Pai et al., 2017; Cui et al., 2018). Targeting β-catenin signaling has been proposed as a promising strategy to develop effective anticancer agents (Qin et al., 2018b; Cheng et al., 2019). Recent advances in understanding the protein structures of β-catenin alone and complexed with its coactivators have promoted the design and development of specific small-molecule inhibitors (Krishnamurthy and Kurzrock, 2018; Zhang X. et al., 2020). These β-catenin signaling inhibitors have shown anticancer efficacy in preclinical settings, and some of them have entered clinical trials, such as PRI-724 (Krishnamurthy and Kurzrock, 2018). However, none of these β-catenin inhibitors has been approved for clinical use yet. It is still urgently needed to identify more specific, safe, and effective β-catenin inhibitors for cancer treatment.

Natural products and their derivatives represent a major source for anticancer drug discovery (Qian et al., 2013; Qin et al., 2017). Over the past few decades, about 33.5% of FDA-approved anticancer drug entities are identified from natural products or their derivatives (Newman and Cragg, 2020). Many natural products have been found to exert their anticancer activity by inhibiting oncoproteins (e.g. β-catenin and MDM2) and/or reactivating tumor suppressors (e.g. p53 and Puma) (Li et al., 2013; Qin et al., 2018a; Wang W. et al., 2018; Wang et al., 2020; Zhang J. et al., 2020). It has also been reported that natural products can enhance the chemosensitivity of cancer cells by suppressing the functions of drug resistance-related proteins (Feng et al., 2017; Dong et al., 2020; Yuan et al., 2020). Recent studies have identified several natural products with potent inhibitory effects on the β-catenin signaling and shown promising anticancer efficacy *in vitro* and *in vivo*, such as baicalin (Zhou et al., 2017), arctigenin (Lee et al., 2017), and rhein (Liu et al., 2018). In the present review, we comprehensively discuss the natural products that target the β-catenin signaling, their *in vitro* and *in vivo* anticancer activities, and underlying molecular mechanisms. Moreover, we summarize known natural-product-based β-catenin-targeting strategies and propose new strategies that may be used to identify more specific and effective β-catenin inhibitors for cancer prevention and therapy.

## Wnt/β-Catenin Signaling Pathway

**Figure 1:**
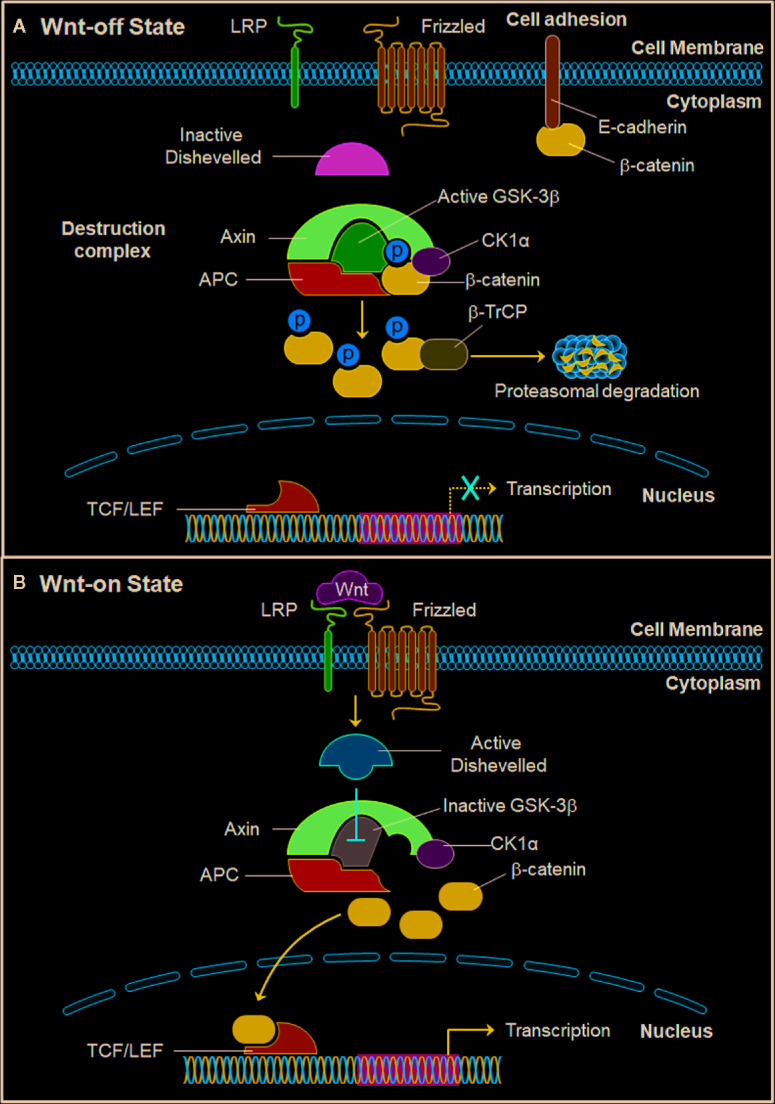
The Wnt/β-catenin signaling pathway. **(A)** In the Wnt-off state, the β-catenin destruction complex is formed by Axin, APC, GSK3β, and CK1α and promotes the phosphorylation of β-catenin. The E3 ligase β-TrCP further induces β-catenin ubiquitination and proteasomal degradation. E-cadherin and β-catenin also form complex to enhance cell adhesion. **(B)** In the Wnt-on state, Wnt proteins bind to Frizzled receptor and LRP co-receptor and recruit and activate Dishevelled, which further inhibits the activity of GSK3β and releases β-catenin from the destruction complex. The stable β-catenin subsequently translocates into the nucleus, interacts with TCF/LEF, and promotes the transcription of its down-stream target genes. APC, adenomatous polyposis coli; β-TrCP, β-Transducin repeat-containing protein; CK1α, casein kinase 1α; GSK3β, glycogen synthase kinase 3β; LRP, low-density lipoprotein receptor-related protein; TCF/LEF, T cell factor/lymphocyte enhancer factor.

The Wnt/β-catenin pathway (**Figure 1**) plays an important role in cancer development and progression by promoting the cytoplasmic accumulation and nuclear translocation of β-catenin and activating the transcription of genes related to cancer cell proliferation, cell cycle progression, anti-apoptosis, migration, invasion, and drug resistance (Krishnamurthy and Kurzrock, 2018). In the absence of Wnt stimulation, β-catenin is phosphorylated by the destruction complex (**Figure 1A**), which includes Axin, adenomatous polyposis coli (APC), glycogen synthase kinase 3 (GSK3), and casein kinase 1α (CK1α) (Stamos and Weis, 2013). When β-catenin is recruited to the destruction complex, CK1α initially phosphorylates β-catenin at Ser45 and GSK3β further promotes β-catenin phosphorylation at Ser33, Ser37, and Thr41 (Amit et al., 2002; Liu et al., 2002; Wu and He, 2006). Subsequently, the phosphorylated β-catenin is recognized and ubiquitinated by the E3 ligase protein β-transducin repeat-containing protein (β-TrCP), which consequently results in the proteasomal degradation of β-catenin (Aberle et al., 1997; Orford et al., 1997; Stamos and Weis, 2013).

When the Wnt ligands bind to the cysteine-rich domain (CRD) of Frizzled (FZD) receptors (**Figure 1B**), the FZD co-receptors LRP5/6 (low-density lipoprotein receptor-related protein 5/6) will be recruited to form complexes with FZD and then phosphorylated, which further causes the disassembly of the destruction complex and the stabilization and accumulation of β-catenin in the cytoplasm (Cheng et al., 2019; Zhang X. et al., 2020). The Wnt/FZD/LRP complex also activates Dishevelled, which further inhibits GSK3β and prevents β-catenin phosphorylation and inactivation (Cheng et al., 2019). The stable β-catenin then translocates into the nucleus and interacts with TCF/LEF to form the β-catenin/TCF/LEF complex, thereby activating the transcription of downstream target genes (Krishnamurthy and Kurzrock, 2018).

## Natural Product Inhibitors of β-Catenin Signaling Pathway

**Figure 2:**
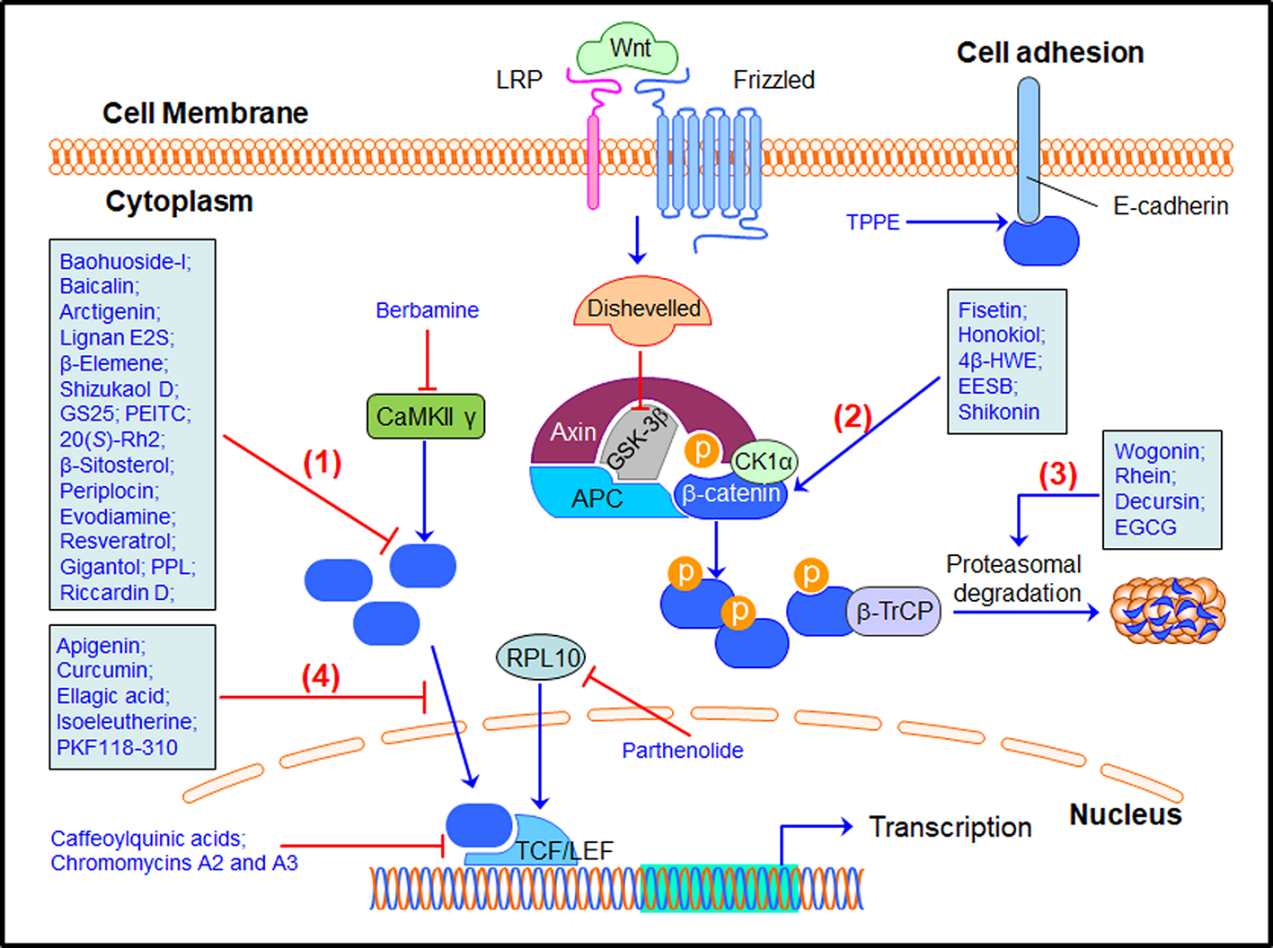
Natural products targeting the β-catenin signaling pathway for cancer therapy. Many natural products have been discovered to inhibit the β-catenin signaling pathway through (1) down-regulating β-catenin expression, (2) modulating β-catenin phosphorylation, (3) promoting β-catenin protein degradation, (4) inhibiting β-catenin nuclear translocation, and other mechanisms of action. 20(*S*)-Rh2, 20(*S*)-ginsenoside Rh2; 4β-HWE, 4β-Hydroxywithanolide E; AEUT, alkaloid-enriched extract of *Uncaria tomentosa*; APC, adenomatous polyposis coli; β-TrCP, β-Transducin repeat-containing protein; CK1α, casein kinase 1α; EGCG, epigallocatechin-3-gallate; EESB, ethanol extract of *Scutellaria barbata* D. Don; GS25, 20(*S*)-25-OCH_3_-PPD; GSK3β, glycogen synthase kinase 3β; LRP, low-density lipoprotein receptor-related protein; PEITC, phenethyl isothiocyanate; PPL, polysaccharides from *Phellinus linteus*; TCF/LEF, T cell factor/lymphocyte enhancer factor; TPPE, *Teucrium polium* plant extract.

As a well-recognized cancer driver gene, β-catenin has been demonstrated as a molecular target for developing anticancer drugs. Small-molecule inhibitors have been designed and synthesized for inhibiting the β-catenin/TCF binding or promoting β-catenin phosphorylation and ubiquitination (Zhang X. et al., 2020). Natural product-based libraries have also been frequently screened, leading to the identification of several β-catenin inhibitors (**Table 1**). These natural products have been found to inhibit the β-catenin signaling through different molecular mechanisms (**Figure 2**), including, but not limited to, 1) down-regulating β-catenin expression, 2) modulating β-catenin phosphorylation, 3) promoting β-catenin protein degradation, 4) inhibiting β-catenin nuclear translocation, and others.

**Table 1:** Natural product β-catenin inhibitors and their activities and mechanisms of action.

Inhibitors	Mechanisms of action	*In vitro* activity	*In vivo* activity	References
***Strategy 1. Down-regulate β-catenin expression***				
Baohuoside-I	Down-regulates the expression of β-catenin and its downstream targets at both mRNA and protein levels	Inhibits cell growth and induces apoptosis in esophageal cancer Eca109 cells	Suppresses tumor growth in mice bearing Eca109 xenograft tumors	(Wang et al., 2011)
Baicalin	Down-regulates β-catenin expression	Inhibits migration and invasion in breast cancer MDA-MB-231 and 4T1 cells	Prevents metastasis to liver and lung in mice bearing 4T1 xenograft tumors	(Zhou et al., 2017)
Arctigenin	Down-regulates β-catenin expression in an ER-dependent manner	Inhibits proliferation and induces apoptosis in ER positive breast cancer MCF7 cells	NR	(Lee et al., 2017)
Lignan E2S	Down-regulates β-catenin expression	Inhibits the viability and induces cell cycle arrest at the G1 phase in colon cancer cells	NR	(Xia et al., 2013)
β-Elemene	Down-regulates the expression of β-catenin and TCF7	Inhibits cell viability, induces cell cycle arrest at G1 phase and apoptosis, and prevents migration and invasion in cervical cancer SiHa cells	NR	(Wang L. t al., 2018)
Shizukaol D	Down-regulates the expression of β-catenin, LRP, Dvl2, and Axin2	Inhibits cell growth and induces apoptosis in liver cancer cells	NR	(Tang et al., 2016)
20(*S*)-25-OCH_3_-PPD	Down-regulates the expression of β-catenin and its downstream targets	Inhibits proliferation and induces apoptosis in colon and lung cancer cells	NR	(Bi et al., 2009)
20(*S*)-ginsenoside Rh2	Down-regulates β-catenin expression at both mRNA and protein levels	Inhibits proliferation and induces cell cycle arrest at G0/G1 phase and apoptosis in leukemia KG−1a cells	NR	(Chen et al., 2016)
β-Sitosterol	Down-regulates β-catenin expression	Inhibit growth and induces apoptosis in colon cancer COLO 320 DM cells	Reduces the number of aberrant crypt and crypt multiplicity in DMH-treated rats	(Baskar et al., 2010)
Periplocin	Down-regulates β-catenin expression	Inhibits the proliferation of colon cancer HCT116 cells	NR	(Kim et al., 2017)
Evodiamine	Down-regulates β-catenin expression	Inhibits proliferation, invasion, and migration of HCC HepG2 and SMMC-7721 cells	Suppresses tumor growth and angiogenesis in mice bearing H22 and SMMC-7721 xenograft tumors	(Shi et al., 2016)
Resveratrol	Down-regulates the expression of β-catenin and c-Myc	Inhibits cell proliferation and induces apoptosis in LNCaP and MES-SA cells	Suppresses tumor growth in mice bearing LNCaP xenograft tumors	(Mitani et al., 2014; Mineda et al., 2019)
PEITC	Reduces the levels of phosphorylated GSK3β and β-catenin	Suppresses the properties of CSCs, inhibits proliferation, and induces apoptosis in colon cancer cells	NR	(Chen et al., 2018)
Gigantol	Reduces the levels of phosphorylated LRP6, total LRP6, and cytosolic β-catenin	Inhibits the viability and migration of breast cancer cells	NR	(Yu et al., 2018)
Polysaccharide from *Phellinus linteus*	Down-regulates the expression of β-catenin and cyclin D1	Inhibits growth, invasion, and motility of colon cancer SW480 cells	Suppresses tumor growth in mice bearing SW480 xenograft tumors	(Song et al., 2011)
Riccardin D	Down-regulates the expression of β-catenin and cyclin D1	NR	Prevents intestinal adenoma formation in *APC^Min/+^* mice	(Liu et al., 2012)
***Strategy 2. Modulate β-catenin phosphorylation***				
Fisetin	Induces β-catenin phosphorylation and inhibits the expression and nuclear translocation of TCF1 and TCF4	Inhibits viability and induces apoptosis in colon cancer HCT116 and HT29 cells	NR	(Suh et al., 2009)
Honokiol	Induces β-catenin phosphorylation at Ser^45^, Ser^33/37^ and Thr^41^	Inhibits the migration of NSCLC cells	NR	(Singh and Katiyar, 2013b)
4β-HWE	Induces β-catenin phosphorylation at Ser^33^, Ser^37^, and Thr^41^ and inhibits its nuclear translocation	Inhibits proliferation and induces cell cycle arrest at G0/G1 phase and apoptosis in colon cancer cells	Suppresses tumor growth in mice bearing HCT116 xenograft tumors	(Ye et al., 2019)
Ethanol extract of *Scutellaria barbata* D. Don	Induces β-catenin phosphorylation and reduces β-catenin expression	Inhibits the proliferation and survival of HT-29 cells	Suppresses tumor growth in mice bearing HT-29 xenograft tumors	(Wei et al., 2017)
Shikonin	Inhibits β-catenin phosphorylation at Y333	Inhibits proliferation, migration, and invasion of glioblastoma U87 and U251 cells	NR	(Zhang et al., 2015)
***Strategy 3. Promote β-catenin protein degradation***				
Wogonin	Promotes β-catenin phosphorylation and degradation	Inhibits proliferation and induces cell cycle arrest at G1 phase in colon cancer cells	Suppresses tumor growth in mice bearing HCT116 xenograft tumors	(He et al., 2013)
Rhein	Promotes β-catenin protein degradation in GSK3-dependent manner	Inhibits proliferation and induces cell cycle arrest at S phase in HepG2 and Hela cells	Suppresses tumor growth in mice bearing HepG2 xenograft tumors	(Liu et al., 2018)
Decursin	Promotes β-catenin protein degradation	Inhibits the proliferation of prostate cancer PC3 cells	NR	(Song et al., 2007)
EGCG	Promotes β-catenin phosphorylation and degradation in a β-TrCP-dependent manner	Suppresses the properties of CSCs, inhibits proliferation, and induces apoptosis in colon cancer cells	NR	(Dashwood et al., 2005; Singh and Katiyar, 2013a; Oh et al., 2014; Chen et al., 2017)
***Strategy 4. Inhibit β-catenin nuclear translocation***				
Apigenin	Inhibits β-catenin nuclear translocation	Inhibits proliferation and induces apoptosis in prostate cancer DU145 cells	Prevents prostate tumorigenesis and metastasis and improves overall survival in TRAMP mice	(Shukla et al., 2007)
Curcumin	Inhibits β-catenin nuclear translocation and the β-catenin/TCF-LEF binding to the promoter DNA	Inhibits the viability, migration, and invasion and induces cell cycle arrest at G2/M phase and apoptosis in osteosarcoma and colon cancer cells	NR	(Jaiswal et al., 2002; Leow et al., 2010)
Ellagic acid	Inhibits β-catenin nuclear translocation	NR	Suppresses development of carcinomas in the DMBA-induced HBP carcinogenesis model.	(Anitha et al., 2013)
Isoeleutherine	Inhibits β-catenin nuclear translocation and TCF/β-catenin transcription	Inhibits the viability of colon cancer cells	NR	(Li et al., 2009)
Toxoflavin (PKF118-310)	Inhibits β-catenin nuclear translocation	Inhibits the viability, migration, and invasion and induces cell cycle arrest at G2/M phase and apoptosis in osteosarcoma cells	NR	(Leow et al., 2010)
***Strategy 5. Others***				
Parthenolide	Binds to RPL10, decreases TCF4/LEF1 protein levels by blocking protein synthesis, and inhibits Wnt/β-catenin signaling pathway	Inhibits proliferation of colon cancer cells	NR	(Zhu et al., 2018)
Berbamine	Decrease β-catenin protein levels by binding to CaMKII γ and inhibiting its kinase activity	Induces apoptotic and autophagic death of CML cells	Suppresses tumor growth in mice bearing TKI-resistant K562 or primary CML xenograft tumors	(Gu et al., 2012)
*Teucrium polium* plant extract	Enhances the formation of E-cadherin/β-catenin complex and inhibits β-catenin phosphorylation	Inhibits proliferation, induces cell cycle arrest at S phase, and reduces invasion and motility of prostate cancer PC3 and DU145 cells	NR	(Kandouz et al., 2010)
Alkaloid-enriched extract of *Uncaria tomentosa*	Inhibits Wnt/β-catenin signaling pathway without affecting β-catenin level	Inhibits viability of colon cancer cells	NR	(Gurrola-Diaz et al., 2011)
Dinactin	Inhibits Wnt/β-catenin signaling pathway	Inhibits the proliferation and induces cell cycle arrest at G1/S phase in HCT116 and HepG2 cells	NR	(Hussain et al., 2019)
Caffeoylquinic acids	Inhibits TCF-4 expression	Inhibits the viability of colon cancer HCT116 cells	NR	(Taira et al., 2014)
Chromomycins A2 and A3	Inhibits TCF/β-catenin transcription	Inhibits the viability of gastric adenocarcinoma cells	NR	(Toume et al., 2014)

4β-HWE, 4β-hydroxy-withanolide E; CML, chronic myeloid leukemia; CSCs, cancer stem cells; DMBA, 7,12-dimethylbenz[a]anthracene; DMH, 1,2-dimethylhydrazine; EGCG, epigallocatechin-3-gallate; EMT, epithelial-to-mesenchymal transition; ER, estrogen receptor; HBP, hamster buccal pouch; HCC, hepatocellular carcinoma; NR, not reported; NSCLC, non-small cell lung cancer; PEITC, phenethyl isothiocyanate; TKI, tyrosine kinase inhibitor.

### Down-Regulate β-Catenin Expression

The majority of natural product inhibitors have been reported to down-regulate the expression of β-catenin and/or its coactivators, thereby inhibiting the transcription of its downstream target genes (**Table 1**). Flavonoids are a class of ubiquitous polyphenolic natural products in medicinal plants and have recently been found to exert anticancer activities by targeting the β-catenin signaling (Cheng et al., 2011b; Wang et al., 2011; Zhu et al., 2012a; Zhou et al., 2017). Baohuoside-I, a flavonoid from *Cortex periplocae* has been reported to down-regulate the expression of β-catenin and its downstream target gene Survivin and Cyclin D1 at both protein and mRNA levels (Wang et al., 2011). Wang et al. have also demonstrated that baohuoside-I inhibits the viability and induces apoptosis in esophageal cancer Eca109 cells *in vitro* and suppresses the growth of Eca109 xenograft tumors *in vivo* (Wang et al., 2011). Another flavonoid baicalin has been found to inhibit the migration and invasion of triple negative breast cancer MDA-MB-231 and 4T1 cells *in vitro* by modulating the expression of epithelial-to-mesenchymal transition (EMT)-related proteins, including β-catenin (Zhou et al., 2017). Further studies have shown that baicalin prevents tumor metastasis to liver and lungs in mice bearing 4T1 xenograft tumors (Zhou et al., 2017). Importantly, the expression level of β-catenin is critical for the anti-metastatic activity of baicalin (Zhou et al., 2017). However, the detailed molecular mechanisms for their inhibitory effects on β-catenin expression are yet to be determined.

Lignans as a group of diphenolic natural products have exhibited inhibitory effects on β-catenin expression (Cheng et al., 2011a; Zhu et al., 2012b; Xia et al., 2013; Lee et al., 2017). Lee et al. have recently demonstrated that β-catenin is a molecular target of the lignan arctigenin (Lee et al., 2017). It has been found that arctigenin inhibits the proliferation of breast cancer MCF7 cells and induces apoptosis *in vitro* in an estrogen receptor (ER)-dependent manner (Lee et al., 2017). Arctigenin has also been shown to decrease the expression levels of β-catenin and its target Cyclin D1 *via* an ER-dependent mechanism (Lee et al., 2017). The lignan E2S from *Carya cathayensis* fruits has also shown inhibitory effects on β-catenin expression (Xia et al., 2013). Xia et al. have reported that the lignan E2S inhibits cell viability and induces cell cycle arrest at the G1 phase in colon cancer HT29, HCT116, LoVo, and SW480 cells in a concentration-dependent manner (Xia et al., 2013). However, their *in vivo* efficacy and mechanisms of action should be further investigated.

Sesquiterpenes, especially sesquiterpene lactones are a class of natural products with diverse structures and biological activities (Zhang et al., 2012; Wang et al., 2014). Wang *et al.* have recently reported that the sesquiterpene β-elemene from *Rhizoma zedoariae* oil inhibits cell viability and induces cell cycle arrest at the G1 phase and apoptosis in cervical cancer SiHa cells (Wang L. t al., 2018). β-Elemene has also been shown to suppress the migration and invasion of SiHa cells (Wang L. t al., 2018). The mechanisms of action studies have found that β-elemene inhibits the β-catenin signaling by decreasing the expression levels of β-catenin and its coactivator TCF7 (Wang L. t al., 2018). The dimeric sesquiterpenes have shown more potent anticancer activities than their monomers by targeting oncogenic drivers (Qin et al., 2015a; Qin et al., 2015b). Tang et al. have recently identified shizukaol D, a dimeric sesquiterpene as a β-catenin signaling inhibitor (Tang et al., 2016). It has been found that shizukaol D inhibits cell viability and colony formation and induces apoptosis in liver cancer cells by down-regulating the expression of β-catenin and its upstream regulators LRP, Dvl2, and Axin2 (Tang et al., 2016). The activation of β-catenin by wnt3a blocks shizukaol D-induced cell growth inhibition, indicating that the β-catenin signaling plays a critical role in the anticancer activity of this compound (Tang et al., 2016). However, the *in vivo* efficacy and safety of β-elemene and shizukaol D have not been evaluated yet. It should also be examined whether these sesquiterpenes induce β-catenin degradation by disrupting the destruction complex.

Ginsenosides, the active components of the well-known herbal medicine *Panax ginseng* have shown a wide range of pharmacological activities, including anticancer activity (Nag et al., 2012). Recent studies have indicated that β-catenin signaling also plays an important role in the anticancer activities of ginsenosides (Bi et al., 2009; Chen et al., 2016). Bi et al. have found that ginsenoside 20(*S*)-25-OCH_3_-PPD inhibits the viability and induces apoptosis in colon and lung cancer cells by downregulating the protein expression of β-catenin and its targets CDK4, cyclin D1, c-Myc, and TCF4 (Bi et al., 2009). More recently, Chen et al. have reported that 20(*S*)-ginsenoside Rh2 inhibits cell viability and induces cell cycle arrest at the G0/G1 phase and apoptosis in leukemia KG−1a cells by down-regulating the expression of β-catenin at both protein and mRNA levels (Chen et al., 2016). Nevertheless, further studies should be performed to explore the precise mechanisms of action for the ginsenoside-mediated inhibition of β-catenin expression.

Sterols from medicinal plants have recently been shown to inhibit the β-catenin signaling (Baskar et al., 2010; Kim et al., 2017). Baskar et al. have found that β-sitosterol, a sterol compound from the traditional medicinal plant *Asclepias curassavica* Linn. inhibits cell growth and induces apoptosis in colon cancer COLO 320 DM cells (Baskar et al., 2010). β-Sitosterol has also shown chemopreventive effects on 1,2-dimethylhydrazine (DMH)-induced colon carcinogenesis in rats (Baskar et al., 2010). The *in vitro* and *in vivo* anti-colon cancer activity of β-sitosterol has been partially attributed to the down-regulation of β-catenin expression by the compound (Baskar et al., 2010). Kim *et al.* have identified periplocin from the methanol extract of *Telectadium dongnaiense* bark as a new inhibitor of the β-catenin signaling (Kim et al., 2017). It has been reported that periplocin decreases the protein expression levels of β-catenin and its downstream targets, leading to the growth inhibition of colon cancer HCT116, SW480, HCT15, and LS174T cells (Kim et al., 2017). Nature-derived alkaloids have also exhibited potent anticancer activity by targeting the β-catenin signaling (Fu et al., 2011; Shi et al., 2016). Evodiamine, a quinolone alkaloid from *Euodia rutaecarpa* (Juss.) Benth. (Rutaceae) has been reported to inhibit the proliferation, invasion, and migration of hepatocellular carcinoma (HCC) HepG2 and SMMC-7721 cells *in vitro* and suppress tumor growth and angiogenesis in mice bearing H22 and SMMC-7721 xenograft tumors *in vivo* (Shi et al., 2016). The down-regulation of β-catenin protein expression by evodiamine has been found to contribute to its anti-HCC activity (Shi et al., 2016).

Several other natural products have been found to down-regulate the expression of β-catenin, including resveratrol (Mitani et al., 2014; Mineda et al., 2019), phenethyl isothiocyanate (PEITC) (Chen et al., 2018), gigantol (Yu et al., 2018), polysaccharide from *Phellinus linteus* (Song et al., 2011), and riccardin D (Liu et al., 2012). Among them, resveratrol and PEITC are two well-documented dietary anticancer chemopreventive compounds with multiple molecular targets (Mitani et al., 2014; Chen et al., 2018; Mineda et al., 2019). Recent studies have shown that resveratrol and PEITC also inhibit the β-catenin signaling; however, the importance of β-catenin in their anticancer activity is yet to be determined (Mitani et al., 2014; Chen et al., 2018; Mineda et al., 2019). Besides, gigantol from medicinal orchids and polysaccharides from *Phellinus linteus* have shown inhibitory effects on cancer cell growth and invasion by decreasing the protein levels of β-catenin and its targets (Song et al., 2011; Yu et al., 2018). Moreover, riccardin D from the liverwort plant *Dumortiera hirsute* has shown preventive effects on intestinal adenoma formation in *APC* ^Min/+^ mice (Liu et al., 2012). Although riccardin D has been shown to decrease the protein expression level of β-catenin *in vivo*, it is unknown how important β-catenin is in its anticancer activity (Liu et al., 2012).

### Modulate β-Catenin Phosphorylation

Considering that β-catenin phosphorylation by GSK3β and CK1α leads to its inactivation and degradation, directly promoting β-catenin phosphorylation has been proposed as an effective strategy to inhibit the β-catenin signaling pathway. Several natural products have recently been shown to inhibit cancer cell growth and metastasis by inducing β-catenin phosphorylation and inactivation (**Table 1**). Suh *et al.* have reported that fisetin, a flavonoid commonly found in various vegetables and fruits promotes the phosphorylation of β-catenin and decreases its nuclear level in colon cancer cells (Suh et al., 2009). It has also been found that fisetin down-regulates the expression of TCF1 and TCF4 in the nucleus and whole cancer cells (Suh et al., 2009). Consequently, fisetin inhibits cell viability and induces apoptosis in colon cancer HCT116 and HT29 cells (Suh et al., 2009). Honokiol, a lignan from the bark of *Magnolia* plants has been found to induce the phosphorylation of β-catenin at Ser45, Ser33/37, and Thr41 and reduce the β-catenin protein level in the nucleus (Singh and Katiyar, 2013b). Singh and Katiyar have also demonstrated that honokiol suppresses the migration of non-small cell lung cancer (NSCLC) cells (Singh and Katiyar, 2013b). However, the effects of fisetin and honokiol on the protein stability of β-catenin are yet to be determined. Moreover, the *in vivo* efficacy of both compounds should be examined in future studies.

Ye *et al.* have recently reported that 4β-hydroxywithanolide E (4β-HWE), a natural withanolide from *Physalis peruviana* increases the level of phosphorylated β-catenin and decreases the levels of active nonphosphorylated form and total β-catenin in colon cancer HCT116 cells (Ye et al., 2019). 4β-HWE has been shown to inhibit cell viability and induce cell cycle arrest at the G0/G1 phase and apoptosis in colon cancer cells with minimal cytotoxicity against normal colonic epithelial cells (CCD-841-CoN) (Ye et al., 2019). More importantly, this compound has shown potent inhibitory effects on tumor growth in mice bearing HCT116 xenograft tumors, without causing significant changes in the average body weights of mice (Ye et al., 2019). Wei *et al.* have found that ethanol extract of *Scutellaria barbata* D. Don (EESB) inhibits the viability and proliferation of colon cancer HT-29 cells *in vitro* and suppresses the growth of HT-29 xenograft tumors *in vivo* (Wei et al., 2017). Mechanism of action studies have indicated that EESB induces β-catenin phosphorylation and reduces the expression level of total β-catenin in HT-29 cells (Wei et al., 2017). However, the active components in EESB that are responsible for EESB-induced β-catenin phosphorylation are yet to be determined.

Different from the aforementioned natural products that induce β-catenin phosphorylation, shikonin, an anthraquinone derivative from the root of *lithospermum* inhibits the phosphorylation of β-catenin in glioblastoma cells in a context-dependent manner. More specifically, this compound inhibits β-catenin phosphorylation at Tyr333 in U87 cells without altering its level in U251 cells (Zhang et al., 2015). However, shikonin has exhibited inhibitory effects on the proliferation, migration, and invasion of both U87 and U251 cell lines (Zhang et al., 2015). Further studies have indicated that shikonin also inhibits the PI3K/Akt pathway, which may also play a pivotal role in its anticancer activity (Zhang et al., 2015).

### Promote β-Catenin Protein Degradation

Natural products that promote β-catenin protein degradation have been identified and shown anticancer efficacy *in vitro* and *in vivo* (**Table 1**) (Dashwood et al., 2005; Song et al., 2007; He et al., 2013; Liu et al., 2018). He et al. have characterized wogonin, a flavonoid from a Chinese medicinal herb *Scutellaria radix* (also called Huang-Qin) as an inducer of β-catenin protein degradation (He et al., 2013). It has been found that wogonin inhibits cell viability and colony formation and induces cell cycle arrest at the G1 phase in colon cancer cells *in vitro*. Wogonin has also been shown to suppress the growth of HCT116 xenograft tumors *in vivo* (He et al., 2013). Mechanistically, wogonin promotes β-catenin phosphorylation and degradation by activating the destruction complex proteins GSK3β and Axin. It has further been found that wogonin inhibits CDK8 activity, which is at least partially responsible for the inhibition of β-catenin signaling by the compound (He et al., 2013). Rhein, an anthraquinone derivative of rhubarb has also been shown to promote β-catenin phosphorylation at Ser33 and protein degradation, in which GSK3β plays a critical role (Liu et al., 2018). Liu *et al.* have further found that rhein inhibits cell proliferation and induces cell cycle arrest at the S phase in HepG2 and Hela cells *in vitro* and suppresses the growth of HepG2 xenograft tumors *in vivo* (Liu et al., 2018).

Decursin, a pyranocoumarin identified from the roots of *Angelica gigas* Nakai has exhibited promising anti-prostate cancer efficacy (Song et al., 2007). Song *et al.* have recently screened and identified decursin as an inhibitor of the β-catenin signaling using HEK293 cells overexpressing β-catenin/TCF reporter gene (Song et al., 2007). Further studies have shown that decursin promotes the proteasomal degradation of β-catenin in a β-TrCP-dependent but GSK3β-independent manner (Song et al., 2007). Epigallocatechin-3-gallate (EGCG), the most abundant polyphenol from green tea has also been characterized as an inducer of β-catenin protein degradation. Dashwood *et al.* have initially found that EGCG inhibits the β-catenin signaling by decreasing β-catenin protein levels in the nucleus, cytoplasm, and membrane-associated fraction (Dashwood et al., 2005). Moreover, EGCG has been shown to facilitate the trafficking of β-catenin into lysosomes, which contributes to the inhibition of β-catenin signaling (Dashwood et al., 2005). The inhibitory effects of EGCG on β-catenin signaling have further been confirmed in skin and colon cancer cells (Singh and Katiyar, 2013a; Oh et al., 2014; Chen et al., 2017). Oh et al. have reported that EGCG promotes β-catenin phosphorylation at Ser45 and Ser33/37 and proteasomal degradation in a β-TrCP-dependent manner (Oh et al., 2014). Chen *et al.* have shown that EGCG suppresses the properties of cancer stem cells (CSCs), inhibits proliferation, and induces apoptosis in colon cancer cells (Chen et al., 2017).

### Inhibit β-Catenin Nuclear Translocation

β-Catenin acts as a coactivator for TCF/LEF-mediated transcription when the cytoplasmic β-catenin enters the nucleus and forms the β-catenin/TCF/LEF complex. Several natural products have been demonstrated to inhibit the nuclear translocation of β-catenin (**Table 1**), thereby inhibiting cancer cell growth and inducing apoptosis (Jaiswal et al., 2002; Shukla et al., 2007; Li et al., 2009; Leow et al., 2010; Anitha et al., 2013). Shukla *et al.* have reported that apigenin, a plant flavonoid has potent chemopreventive efficacy in TRAMP mice by preventing prostate tumorigenesis and metastasis and improving their overall survival (Shukla et al., 2007). Further studies have shown that apigenin decreases the nuclear level of β-catenin and increases the cytoplasmic level of E-cadherin in prostate cancer DU145 cells *in vitro* and in prostate tumors from TRAMP mice *in vivo* (Shukla et al., 2007). The well-known chemopreventive agent curcumin, a dietary polyphenol from the ginger family *Curcuma longa* has been shown to down-regulate the nuclear level of β-catenin and disrupt the binding of β-catenin/TCF/LEF to the promoter DNA, thus blocking β-catenin-dependent gene expression, inhibiting cell viability, migration, and invasion, and inducing cell cycle arrest at the G2/M phase and apoptosis in osteosarcoma and colon cancer cells (Jaiswal et al., 2002; Leow et al., 2010). Jaiswal *et al.* have also found that curcumin promotes caspase-3-mediated degradation of β-catenin, E-cadherin, and APC, which may cause the loss of cell-cell adhesion (Jaiswal et al., 2002).

Ellagic acid, a nature-derived polyphenol has been found to suppress the development of oral carcinomas in the 7,12-dimethylbenz[a]anthracene-induced hamster buccal pouch carcinogenesis model (Anitha et al., 2013). Its anticancer efficacy has been attributed to the inhibition of β-catenin nuclear translocation, while inactivation of NF-κB by ellagic acid may contribute to the inhibition of the β-catenin signaling (Anitha et al., 2013). In addition, isoeleutherine and toxoflavin (PKF118-310) have been shown to decrease the nuclear accumulation of β-catenin without affecting its cytoplasmic expression level (Li et al., 2009; Leow et al., 2010). PKF118-310 has also been found to inhibit the viability, migration, and invasion and induce cell cycle arrest at the G2/M phase and apoptosis in osteosarcoma U2OS cells (Leow et al., 2010).

### Others

Several natural products have been reported to inhibit the β-catenin signaling by targeting the upstream regulators without direct effects on β-catenin itself (**Table 1**). Zhu et al. have recently identified a plant-derived sesquiterpene lactone, parthenolide as a small-molecule inhibitor of the β-catenin signaling through a high-throughput screening (Zhu et al., 2018). It has further been found that parthenolide directly binds to the ribosome protein RPL10, blocks the protein synthesis of TCF4/LEF1, and decreases their protein levels, therefore inhibiting β-catenin/TCF/LEF-mediated gene transcription and the proliferation of colon cancer cells, without affecting the stability and subcellular distribution of β-catenin (Zhu et al., 2018). Gu et al. have found that berbamine, an alkaloid from traditional Chinese medicine *Berberis amurensis* specifically binds to the ATP-binding pocket of CaMKII γ and inhibits its kinase activity, thereby inhibiting its downstream targets, including β-catenin (Gu et al., 2012). It has been further found that berbamine induces apoptotic and autophagic death of leukemia cells *in vitro* and suppresses tumor growth in mice bearing tyrosine kinase inhibitors (TKI)-resistant K562 or primary chronic myeloid leukemia (CML) xenograft tumors *in vivo* (Gu et al., 2012).

Natural products that restore the E-cadherin/β-catenin complex have been found to prevent cancer metastasis (**Table 1**) (Kandouz et al., 2010). Kandouz *et al.* have found that *Teucrium polium* plant extract (TPPE) enhances the formation of E-cadherin/β-catenin complex, inhibits β-catenin phosphorylation, and reduces invasion and motility of prostate cancer PC3 and DU145 cells (Kandouz et al., 2010). TPPE has also been shown to inhibit prostate cancer cell proliferation and induce cell cycle arrest at the S phase (Kandouz et al., 2010). In addition, several other natural products (**Table 1**), including alkaloid-enriched extract of *Uncaria tomentosa*, dinactin, caffeoylquinic acids, and chromomycins A2 and A3 have shown inhibitory effects on the β-catenin signaling in cancer cells; however, their detailed mechanisms of action are not clear yet (Gurrola-Diaz et al., 2011; Taira et al., 2014; Toume et al., 2014; Hussain et al., 2019).

## Conclusions and Future Perspectives

Thanks to the substantial advances in understanding the molecular basis of cancer initiation, progression, metastasis, and drug resistance, several promising molecular targets have been characterized for cancer drug discovery, including β-catenin (Cui et al., 2018; Qi et al., 2020). Considering the critical role of β-catenin signaling in cancer development and progression, several targeting strategies have been developed to inhibit β-catenin, resulting in the identification of various types of β-catenin inhibitors (Krishnamurthy and Kurzrock, 2018; Qin et al., 2018b). Natural products and natural product-derived compounds remain an important source for the discovery and development of new anticancer drugs (Qian et al., 2013; Qin et al., 2018a; Davison and Brimble, 2019). Several natural products have been shown to inhibit the β-catenin signaling *via* different molecular mechanisms, including, but not limited to, 1) down-regulating β-catenin expression, 2) modulating β-catenin phosphorylation and inducing its inactivation, 3) promoting β-catenin protein ubiquitination and proteasomal degradation, 4) inhibiting β-catenin nuclear translocation, and 5) other.

The majority of natural product β-catenin inhibitors have been shown to down-regulate β-catenin expression at protein and/or mRNA levels and suppress cancer cell growth *in vitro* and *in vivo*. However, the detailed molecular mechanisms for the down-regulation of β-catenin expression by these natural products are not clear yet, which largely hinders the further development of these natural products as anticancer agents. Of note, simply reducing the expression of an oncogene may not only induce cancer cell death but also cause other adverse effects (Wang et al., 2019). It has been found that β-catenin interacts with E-cadherin to stabilize cell-cell adhesion and prevent metastasis (Huber and Weis, 2001). Therefore, down-regulating the expression of membrane-bound β-catenin may promote metastasis, while targeting active β-catenin without affecting the β-catenin/E-cadherin complex is critical for developing safe and effective anticancer agent.

Inhibiting active β-catenin through modulating β-catenin phosphorylation and promoting its proteasomal degradation in the cytoplasm and/or inhibiting the translocation of β-catenin from cytoplasm to nucleus is more promising than reducing the level of β-catenin in whole cells. Although several natural products have been identified to inhibit the β-catenin signaling through these mechanisms of action, it is still unclear whether β-catenin is the real molecular target of these natural products. Therefore, the precise mechanisms of action, especially binding mechanisms should be further investigated. If the binding of these natural products to β-catenin is confirmed, it may be considered to develop β-catenin PROTACs (proteolysis targeting chimeras) by using these compounds for cancer prevention and therapy (Neklesa et al., 2017). Moreover, further evaluation of these compounds in more clinically relevant cancer models should be performed in the future.

## Author Contributions

J-JQ conceptualized the manuscript. W-KY, Z-YX, LY, SM, BX, and X-DC collected the literature, wrote the manuscript and made the figures. J-JQ edited and made significant revisions to the manuscript. All authors contributed to the article and approved the submitted version.

## Funding

This study was supported by National Natural Science Foundation of China (81903842), Program of Zhejiang Provincial TCM Sci-tech Plan (2020ZZ005), and Zhejiang Chinese Medical University Startup Funding (111100E014).

## Conflict of Interest

The authors declare that the research was conducted in the absence of any commercial or financial relationships that could be construed as a potential conflict of interest.
